# Evaluating the Efficacy of a Web-Based Program (Diapason) for Informal Caregivers of Patients With Alzheimer’s Disease: Protocol for a Randomized Clinical Trial

**DOI:** 10.2196/resprot.2978

**Published:** 2013-12-06

**Authors:** Victoria Cristancho-Lacroix, Hélène Kerhervé, Jocelyne de Rotrou, Alexandra Rouquette, Grégory Legouverneur, Anne-Sophie Rigaud

**Affiliations:** ^1^Broca Hospital-APHPDepartment of GerontologyParisFrance; ^2^Research team EA 4468Institut de PsychologieUniversité Paris DescartesParisFrance; ^3^Biostatistic and Epidemiology DepartmentHotel-Dieu HospitalParis-Descartes UniversityParisFrance; ^4^Research team EA 4468University Paris DescartesParisFrance

**Keywords:** family caregivers, Alzheimer's disease, Internet, program effectiveness, psychoeducational program, psychological stress, randomized clinical trials

## Abstract

**Background:**

Informal caregivers (CGs) of patients with Alzheimer’s disease are at risk of suffering from psychological and physical weakening. Several psychoeducational interventions have been designed to prevent stress and burden of caregivers. In France, despite health authorities’ recommendations, to our knowledge there is no rigorously assessed Web-based psychoeducational program to date.

**Objective:**

The objective of our study was to assess the efficacy of a French Web-based psychoeducational program (called Diapason) with an unblinded randomized clinical trial.

**Methods:**

In this protocol, 80 informal caregivers of patients followed at Broca Hospital are recruited offline and randomized in the experimental condition (EC) or the control condition (CC). The volunteers in EC have to visit a closed online user group at least once a week and validate one new session of this fully automated Web program, during 12 weeks. Each week a new thematic is added to the website. The participants in the CC receive usual care, and have access to the Diapason program after their participation (6 months). Face-to-face evaluations for both groups are planned every 3 months (M0–M3 and M6). The main objective of this program is to provide CGs with information on the disease process, how to prevent psychological strain (using anticipation and relaxation techniques), and offering a virtual space (forum) to discuss with other caregivers. The primary outcome of this study is the self-perceived stress, while self-efficacy, burden, depression, and self-perceived health status are defined as secondary outcomes. Other variables that might have an impact on the program efficacy are collected.

**Results:**

This protocol was accepted for funding. The enrollment began in October 2011, and participants currently recruited will finish their evaluations in January 2014. The results are expected for June 2014.

**Conclusions:**

Findings might provide empirical evidence on: (1) the feasibility of an Internet-based program in the French context, (2) the effectiveness of a Web-based program for informal caregivers, and (3) the identification of caregivers who will benefit from this type of intervention.

**Trial Registration:**

Clinicaltrials.gov NCT01430286; http://clinicaltrials.gov/ct2/show/NCT01430286 (Archived by WebCite at http://www.webcitation/6KxHaRspL).

## Introduction

### Background

Informal caregivers (CGs) of patients with dementia have an important role in the prevention of patients’ institutionalization. Unfortunately, CGs are prone to high levels of stress and are at higher risk of weakening mental and physical health, lower life expectancy, and lesser economic security than people who are not confronted with such stressful situations [1]. In order to prevent these consequences various programs have been developed for them, which have shown a positive effect on caregivers’ burden, depression, or stress [2-4]. Furthermore, several studies have demonstrated the protective role of resilience and coping factors for this population [5].

The new recommendations following French Alzheimer's Plan 2013 [6] underlined the use of Web-based interventions in order to inform and support family caregivers.

### Distance-Based Interventions

There are many reasons for caregivers to use or to prefer a distance intervention instead of a face-to-face one. In fact, CGs spend a lot of their time in care activities, supporting directly (eg, cooking, housekeeping, supervising their loved ones) or indirectly (eg, doing administrative, financial, or logistic management) their relatives. Furthermore, the time requested for caring increases with the disease progression, and finding time for their own respite can be quite difficult. In fact, several CGs fulfill many roles, such as being parent, grandparent, worker, and friend. Finally, some of them live in remote regions and other CGs do not feel at ease with face-to-face interventions or prefer a flexible time/content intervention [7].

Distance interventions, based on information and communications technology (ICT), appeared in the earlier part of the 21st century in order to propose an alternative intervention to caregivers unable to access health centers delivering face-to-face programs. Distant programs have shown a positive effect on self-perceived stress, burden, depression symptoms, and social support of caregivers [7-14]_._

In the case of caregivers of patients with dementia, several websites exist in France, but these programs have not been, to our knowledge, subjected to a randomized clinical trial.

It is therefore relevant to evaluate the impact of ICT-based or distance-based interventions on the mental and physical health status of caregivers in a controlled experimental study with a French population. It could represent a base for the health care policies and facilitate financial support for these initiatives.

*Diapason* [15] is a fully automated Web-based version of a psychoeducational program, inspired by the group intervention sessions from the geriatric service of Broca Hospital called Aide dans la Maladie d’Alzheimer (AIDMA) program, or in English: Help in Alzheimer’s disease. AIDMA was assessed in a previous study including 167 dyads “patient-caregiver” and showed a significant improvement in disease understanding and in the ability to cope with care-recipients’ disease [16,17]. The difficulty to schedule and attend all sessions (once per week during 12 weeks) for some of the caregivers was the main reason to adapt the program into an Internet-delivered version. Thus, we have adapted and designed a Web-based program in order to improve the accessibility for caregivers.

The purpose of this article is to present the study protocol of a randomized clinical trial designed to evaluate the efficacy of Diapason, a Web-based psychoeducational program for caregivers of patients with Alzheimer’s disease (AD). Our hypothesis is that the Diapason program reduces the caregiver’s perceived stress and burden and enhances his/her self-efficacy and self-perceived health. This study protocol has received approval from the French competent authorities (ie, Agence Française de Sécurité Sanitaire des Produits de Santé, Centre de Protection de Personnes–CPP, Commission nationale de l'informatique et des libertés).

## Methods

### Study Design

This is a pragmatic and unblinded randomized controlled trial (NCT01430286) of a Web-based psychoeducational program for the CGs of patients diagnosed with AD. Two parallel groups are compared. The experimental group receives immediate access to a Web-based program, and the comparison group is given the information usually delivered to the patient by the geriatrician during follow-up consultations. In addition to the baseline visit, two follow-up visits at the hospital are planned at 3 and 6 months.

### Participant Eligibility

Eligible participants are informal French-speaking caregivers (family or not, providing care to the patient at least 4 hours per week) of an AD patient diagnosed at the Memory Center of the Broca Hospital, Paris, France, and who meets the criteria in the Diagnostic and Statistical Manual of Mental Disorders, 4th Edition [18] or National Institute of Neurological and Communicative Disorders and Stroke and the Alzheimer’s disease and Related Disorders Association criteria [19]. To be included in the trial, caregivers have to be 18 years or older or to be able to provide an informed consent, to score 12 or over on the Perceived Stress Scale of 14 items (PSS-14) [20] during screening, and to have a computer with an Internet access at home with an email address regularly used. If participants (CGs) are on psychopharmacological treatment or therapy, they are required to keep the same treatment at least two months before inclusion in the protocol.

### Exclusion Criteria

Exclusion criteria include being a professional or paid caregiver, a volunteer suffering from a physical or mental health status incompatible with patient's care, or following another psychoeducational program.

### Recruitment

Strategies to communicate about the program include flyers and posters in medical waiting rooms of the Memory Center as well as in other places in the Broca Hospital. An information meeting for the hospital staff has been organized before starting the inclusions in order to explain the study protocol. Then, the contact forms are available in every counseling room and in the waiting room.

The participants are recruited either during the follow-up consultation of a patient: (1) the geriatrician/neurologist delivers the general information about the protocol and gives a contact form to fill in and drop off at the Memory Center’s reception desk, or (2) the CGs fill in the contact form available in the waiting room and drop it off at the Memory Center’s reception desk.

One of the two research psychologists previously trained in the protocol contacts the caregiver, checks his/her eligibility criteria and explains the benefits, constraints, and schedule of the protocol. The psychologist gives an information notice to the caregiver and proposes to contact him/her a few days later. If the caregiver agrees with the protocol and meets the criteria for inclusion, the screening session (M0) is scheduled with the caregiver.

### Randomization

A computer-generated randomization list is used to assign the participants in the experimental condition (EC) group or in the control condition (CC) group after assessment with PSS-14 and all the inclusion and noninclusion criteria are checked. Blocking and stratification by gender and relationship (spouses versus nonspouses) were used to generate the randomization list.

### Interventions

#### Experimental Condition

The Diapason program is an adapted fully automated computerized version of a psychoeducational program (AIDMA) created by the Geriatric Service of Broca Hospital. Usability of Diapason program was evaluated in a previous experimental study (pre/post). The study involved the assessment of 30 volunteer participants 60 years or older, with various levels of expertise in Internet use, during a guided visit. After modifications and adaptation of the website, the performances of beginners and experts were similar [21].

**Figure 1:**
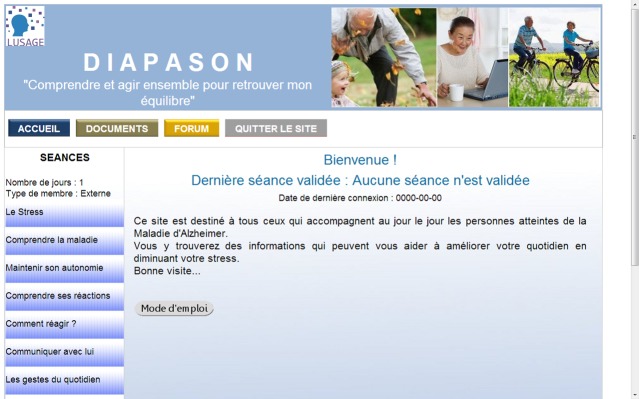
Home page.

Diapason is a free password-protected website. Figure 1 shows the home page. The program is run in twelve thematic weekly sessions organized in the following order: (1) caregiver stress, (2) understanding the disease, (3) maintaining the loved ones’ autonomy, (4) understanding their reactions–how to recognize behavioral and emotional troubles, (5) coping with behavioral and emotional troubles, (6) communicating with loved ones, (7) improving their daily lives, (8) avoiding fall risks, (9) pharmacological and nonpharmacological interventions, (10) social and financial support, (11) about the future, (12) in a nutshell–a summary of Diapason program.

Globally, these twelve sessions cover the following areas: (1) information about AD diagnosis, symptoms, treatment, and progression, (2) how to cope with stressful situations, and (3) information about socioeconomic support and preventive gestures. A new session is available each week, after the validation of the previous session. Furthermore, the website also contains: (1) relaxation guidelines and training videos (based on Schultz’s Autogenic Training and Jacobson’s method) [22,23], (2) stories based on testimonials of caregivers, used to show critical situations and possible solutions to manage them (eg, apathy of patient, caregivers’ isolation), (3) a glossary for the technical words (eg, neuropsychological assessment, aphasia), (4) stimulation guidelines and entertainment activities to do with the patients, and (5) a forum allowing users to establish contact with other caregivers anonymously, express their concerns, discuss solutions to daily problems, and share their feelings and experiences. The participants use nicknames to protect their privacy. A clinical psychologist takes part in discussions if necessary (ie, aggressive or inappropriate comments).

Participants involved in experimental group have to validate one session per week during 12 weeks (about 10 minutes per session), and complete a satisfaction survey corresponding to each session. During the first evaluation (M0) the participant is trained by a psychologist in how to use the website. At the second visit (M3) the participant is requested to provide the satisfaction paper-based survey filled out.

#### Control Condition

Participants randomized in the CC group receive usual care. It consists of a geriatric semiannual follow-up appointment during which the caregiver obtains illness information from the geriatrician. The volunteers receive the access code to the Diapason website at the end of their participation in the research protocol. Every participant of the CC group is advised to look for more specific help (ie, that of a psychologist or a physician) when he/she feels it necessary and then to report it to the main investigator.

### Measures and Procedures

#### Participant Recruitment

The physicians of the Memory Center have been informed on the study protocol and have received training in inclusion criteria screening. They provide the caregiver with some information about this study at the end of the consultation with the patient. Then the physician gives a contact form to the volunteers interested in participating in the study. The research psychologist contacts the person, presents the protocol study, and provides the caregiver with the information sheet. When the participant delivers a positive answer, the first visit (M0) at the hospital is scheduled together with the psychologist.

#### Assessment Protocol

The duration of each visit (M0-M3 and M6) is estimated to 90 minutes. The baseline visit is usually conducted as follows:

1. The research psychologist answers the questions on the information notice and the participant signs the informed consent if he/she agrees.
2. Evaluation with PSS-14 (primary outcome).
3. Randomization if PPS-14 total score is 12 or over.
4. Demographical interview and control questions of caregiver and patient’s variables.
5. Assessment of secondary variables (researcher-administered, and then self-administered surveys).
6. The participants randomized in the EC receive the material (weekly paper-based survey, a journey book, and a user’s manual of the website) and a personal access code to the website. Then, they are trained on how to use the Web-based program.
7. The CC participants are notified that they will receive a website access at the end of their participation to this protocol (6 months after M0).
8. Planning follow-up visits (M3-M6).

For the CC and EC groups, the assessments at M3 and M6 visits are similar, and go as follows: (1) evaluation of caregiver variables (time spent on caregiving, use of respite resources, stressful events, etc) and patient status (hospitalization or other unexpected event occurred in the last three months), (2) measurements with self-administered scales or administered by an interviewer. The measures used in this RCT are summarized in Table 1.

**Table 1:** Overview of measures in the baseline and follow-up visits.

Variables (instruments/measures)		Administration	M0	M3	M6
**Caregivers' measures**					
	Self-perceived stress (PSS-14)	^a^ABI	x	x	x
	Self-efficacy ^b^(RSCS)	ABI	x	x	x
	Caregiver perception of troubles ^c^(RMBPC)	ABI	x	x	x
	Burden ^d^(ZBI)	^e^SA	x	x	x
	Self-reported health ^f^(NHP)	SA	x	x	x
	Depressive symptoms ^g^(BDI-2)	SA	x	x	x
	Knowledge about illness ^h^(VAS)	SA	x	x	x
	The quality of the relationship with the patient (VAS)	SA	x	x	x
	Time spent on caregiving ^i^(RBC)	Interview	x	x	x
	Other sources of stress (ie, work, health status, financial status) (RBC)	Interview	x	x	x
	Respite or social help (ie, psychotherapy, associations, technical help, etc) (RBC)	Interview	x	x	x
	Time and frequency using the program (website statistics)	Website	x	x	x
	Satisfaction towards the program content (weekly paper-based survey filled at home)	Weekly survey (M0-M3 for ^j^EC)	x	x	x
**Patients' measures**					
	Cognitive status ^k^(MMSE)	Medical data	x	-	-
	Degree of dependency ^I^(IADL-RBC)	Interview	x	-	-
	Duration of symptoms (RBC)	Interview	x	-	-

^a^ABI=Administered by the interviewer,^b^RSCS=Revised Scale for Caregiving Self-Efficacy, ^c^RMBPC=Revised Memory and Behavior Problem Checklist, ^d^ZBI=Zarit Burden Interview, ^e^SA=Self-administered,^f^NHP=Nottingham Health Profile, ^g^BDI-2=Beck Depression Inventory-second version, ^h^VAS=Visual Analogical Scale, ^i^RBC=Reported by caregiver, ^j^EC=Experimental condition, ^k^MMSE=Mini-Mental State Examination, ^l^IADL=Instrumental Activities of Daily Living

#### Primary Outcome Measure: PSS-14

Stress perceived by the caregiver is measured by the French version of the Perceived Stress Scale, the version of 14 items from Cohen et al [20], translated into French by Bruchon-Schweitzer in 2002 [24]. The PSS-14 is a widely used self-reported scale evaluating the general appraisal of stress in the last month. It consists of 14 items, with scores ranging from 0 (never) to 4 (very often). This scale has demonstrated a high reliability and validity in several studies [25]. The total score range for this scale is 0-56. Due to numerous roles of caregivers (as mentioned above) and in order to target stress specifically related to a caregiving role, we adapted the instruction of the PSS-14 by proceeding with hetero evaluation and adding the following text in bold: "this scale ask[s] you about your feelings and thoughts about your experience with your relative during the last four weeks." The rest of the instruction is similar to that proposed by Cohen in 1983.

#### Secondary Outcomes Measures Administered by an Interviewer

##### Self-Efficacy

The Revised Scale for Caregiving Self-Efficacy was validated in 2002 by Steffen et al [26] and translated into French by Marziali and Garcia in 2011 [27]. This scale offers a simple and effective way to evaluate caregivers’ self-efficacy on: (1) obtaining respite, (2) controlling upsetting thoughts, and (3) responding to disruptive patient behaviors. Each section has five items arranged from easiest to most difficult (based on research results) [26]. For each item the participants choose a score between 0 and 100, based on their degree of confidence for each situation. This scale should be administered by an interviewer [26].

##### Perceived Behavioral and Cognitive Problems

The Revised Memory and Behavior Problem Checklist [28] is a widely used scale that rates the caregiver’s perceived frequency of occurrence of behavioral and cognitive problems and the caregiver’s perceived distress facing these problems. It explores 24 situations in which the caregiver estimates: (1) the frequency of situations/problems during the last week, and (2) the caregiver’s response to each situation/problem. Satisfactory internal consistency coefficients of reliability have been reported (for frequency of behaviors .93 and for reaction .90) [29].

#### Secondary Outcomes Measures Self-Administered

##### Zarit Burden Interview

The Zarit Burden Interview (ZBI) is a subjective measure of burden that includes 22 items exploring the caregiver’s perception and feelings about care situations. There are three factors that could explain 56.3% of global score variance: (1) caregiver’s social and personal life, (2) psychological burden, and (3) caregiver’s guilt [30]. The score range is 0-88, a higher score indicating a higher burden level.

##### Depressive Symptoms

Depressive symptoms will be evaluated with the second version of Beck Depression Inventory [31]. This widely used scale comprises 21 items, and the total score range is 0-63 [32].

##### Self-Perceived Health

Bucquet et al [33] validated the Nottingham Health Profile in France. We use this scale to evaluate the self-reported morbidity of caregivers. There are 38 items that are grouped in 6 dimensions: (1) physical mobility, (2) social isolation, (3) emotional reactions, (4) pain, (5) sleep, and (6) energy. In the French validation study, weights were calculated using Thurstone's Paired Comparisons [33]. The addition of this item totals a hundred per dimension and corresponds to the percentage of the illness impact perceived by each individual.

#### Additional Measures

##### Caregivers' Measures

The sociodemographic variables and general information on caregiver situation collected are age, sex, educational level, relationship with the patient (spouse versus nonspouse), current psychopharmacological treatment, current psychosocial services and respite care (daycare centers for the patient, in-home care services, etc), time spent per week with the patient, and their “free time”. Moreover, the quality of the relationship with the patient, the caregiver’s confidence in his/her ability to cope with the consequences of the disease, and the caregiver’s level of knowledge about AD are evaluated with the Visual Analogical Scales.

Participants in the EC complete a satisfaction survey each week, after watching the weekly program. Therefore, qualitative information about perceived utility of this program is obtained during the face-to-face interviews in the visits M3 and M6. Moreover, the frequency and duration of the Web-based program use for each participant is stored and anonymously analyzed at the end of the study.

##### Patients' Measures

The global cognitive status of patients is evaluated with the Mini-Mental State Examination (MMSE) [34] and obtained from the patients’ medical file, if the patient accepts it (during follow-up at the Memory Center the patients with AD are evaluated with neuropsychological batteries, including the MMSE evaluation). The degree of dependency from the patient is evaluated by the French version of the Instrumental Activities of Daily Living [35] reported by the caregiver at M0, and the duration of symptoms is also based on the caregivers’ report.

#### Data Management and Statistical Analysis

##### Monitoring/Security Issues

Data are collected via an electronic case-report form, then centralized, and stored on a secured server using the “CleanWEB” system [36]. A monitoring of records is planned every two months and done by an external agent to control the respect of protocol and procedures according to Clinical Best Practices guidelines [37].

##### Ethical Proceedings

This study protocol was submitted to the French ethical CPP and received approval on July 2011. Before they enter the study, all participants receive an information sheet and sign a written consent form.

The study provides equal opportunity to access the program. Caregivers who do not meet the inclusion criteria can access the website and program as external participants. Also, every participant is asked to search another form of help (ie, that of a psychologist or a physician) if he/she feels the need to, and to report it to the main investigator.

##### Sample Size

The sample size has been calculated by the Biostatistics and Epidemiology Department of the Hôtel-Dieu Hospital (Paris). Based on the literature [38], a 6-point difference on PSS-14 scale is expected between EC and CC at the posttest evaluation (M3). With an assumed standard deviation of 9, 40 participants per group should be included to be able to detect such a difference with an 80.0% power (Cronbach alpha=.05; two-tailed).

##### Data Analysis

The Biostatistics and Epidemiology Department of the Hôtel-Dieu Hospital will perform statistical analysis. All the analyses will be conducted according to the intention to treat principle and to handle with missing data; multiple imputations will be used if the missing at random or missing completely at random hypothesis holds. Otherwise sensitivity analysis will be done. No interim analysis will be performed.

A description of the characteristics of the two groups will be performed using percentages for categorical variables and means with standard deviation for quantitative variables. For primary and secondary outcomes, student *t* tests or a Wilcoxon test if required, as well as covariance analysis to take the regression to the mean into account, will be used to compare means between experimental and control groups. Percentages will be compared using chi-square test or Fisher’s exact test if required. Calculations will be performed using SAS software.

Qualitative data obtained during visits M3 and M6 from EG participants’ perception on the program’s utility and satisfaction will be analyzed by Broca’s research team, using thematic analysis [39].

Statistical analysis will exclude data from: (1) caregivers performing less than two thirds of the online program (participant validates fewer than 8 out of 12 sessions), (2) dropouts due to mental or physical state of the caregiver becoming incompatible with this research protocol.

## Discussion

### Distinctive Features

This study protocol is quite innovative. To our knowledge, it is the first French Web-based program evaluated with a randomized clinical trial. The Diapason program has been conceived to offer a primary access to basic information about the illness progression and practical advice to reduce stress and manage the daily life for Alzheimer’s CGs. In coherence with other studies, we are convinced that the Internet for health is an interesting tool to inform and support the isolated CGs [40], at a reduced cost, but with increasing convenience for users [41].

We are interested in evaluating the program effectiveness on self-perceived stress. Although the ZBI is often used to measure the CG stress in the context of dementia [42], the burden construct is relatively complex and not specific enough. In fact, two factor analyses of the ZBI identified three dimensions: (1) personal strain, (2) role strain, and (3) guilt [43], or (1) social impact, (2) psychological burden, and (3) guilt [30]. Based on these results we decided to use a PSS 14-item version and to adapt the main instruction of PSS to caregivers’ strain. However, in this protocol we use the ZBI as a secondary outcome, and it would be interesting to compare the results obtained with each of these measurement instruments.

### Strengths of the Study

In our opinion, four main strengths are identifiable in this protocol.

First, since most elderly caregivers (spouses) do not have sufficient experience with the Internet, 30 elderly volunteers participated in the usability tests, which allowed us to modify and adapt the website prior to the present study. The usability tests increase the likelihood of inexperienced Internet users to use Web-based programs and offers access to a widespread population who has never navigated on the Internet because it was considered as too complex or difficult to use.

Second, our Web-based program (Diapason) keeps a structure that is similar to an on-site psychoeducational program, such that it proposes a thematic session weekly. In this way, we control the information viewed by the caregiver according to a specific schedule. In fact, the EC is not completely controlled if the access to the information is determined by the choice of the patient. In our opinion, controlling the order and access to main thematic areas should improve the reliability of results because all the participants receive the same information.

Third, we are aware of the positive impact of social networking and communication between peers for CGs. We did not have enough human resources to offer a virtual presence or face-to-face participation, nevertheless we integrated a forum in the website which enables the CGs to ask and share experiences, feelings, and advice with their peers, with the participation of a psychologist as moderator This initiative represents a first step towards more comprehensive and interactive Web-based initiatives that our team has scheduled to build, optimizing social networking perspectives, as advised by recent works [44,45].

Fourth, we will analyze the data of Web server utilization from each user and compare it with their satisfaction and appraisal of the effectiveness of the program. This objective information will help us to know the system use, and its acceptability. Our purpose with these results is also to identify the "user profiles" with a highest adherence to or benefit from the program. For instance, do spouses or nonspouses benefit more from the program? Is the time spent on the program website associated with the level of stress after the 12 sessions? Or is there minimum time duration of navigation to observe some benefit from the program?

### Conclusions

In conclusion, the results of this study will allow a better targeting of beneficiaries, for whom the intervention will be more efficient. The results will provide strong support to influence health care policies and facilitate the financial support of these initiatives.

## Abbreviations

AD: Alzheimer’s disease
AIDMA: Aide dans la Maladie d’Alzheimer program
CC: control condition
CGs: Informal caregivers
CPP: Centre de Protection de Personnes
EC: experimental condition
ICT: information and communications technology
MO: month 0
M3: month 3
M6: month 6
MMSE: Mini-Mental State Examination
PSS-14: Perceived Stress Scale of 14 items
ZBI: Zarit Burden Interview

## Multimedia Appendix 1

CONSORT-EHEALTH checklist V1.6.2 [46].

## References

[1] Vitaliano PP, Zhang J, Scanlan JM (2003). Is caregiving hazardous to one's physical health? A meta-analysis. Psychol Bull.

[2] Beauchamp N, Irvine AB, Seeley J, Johnson B (2005). Worksite-based Internet multimedia program for family caregivers of persons with dementia. Gerontologist.

[3] Pinquart M, Sörensen S (2006). Helping caregivers of persons with dementia: Which interventions work and how large are their effects?. Int Psychogeriatr.

[4] Rigaud AS, Pino M, Wu YH, De Rotrou J, Boulay M, Seux ML, Hugonot-Diener L, De Sant'anna M, Moulin F, Legouverneur G, Cristancho-Lacroix V, Lenoir H (2011). Support for patients with Alzheimer's disease and their caregivers by gerontechnology. Geriatr Psychol Neuropsychiatr Vieil.

[5] Harmell AL, Chattillion EA, Roepke SK, Mausbach BT (2011). A review of the psychobiology of dementia caregiving: A focus on resilience factors. Curr Psychiatry Rep.

[6] Ankri J, Van Broeckhoven C.

[7] Benefield LE, Beck C (2007). Reducing the distance in distance-caregiving by technology innovation. Clin Interv Aging.

[8] Powell J, Chiu T, Eysenbach G (2008). A systematic review of networked technologies supporting carers of people with dementia. J Telemed Telecare.

[9] Gallagher-Thompson D, Steffen A, Thompson L (2008). Reducing psychosocial distress in family caregivers. Handbook of behavioral and cognitive therapies with older adults.

[10] Gant JR, Steffen AM, Lauderdale SA (2007). Comparative outcomes of two distance-based interventions for male caregivers of family members with dementia. Am J Alzheimers Dis Other Demen.

[11] Davis JD, Tremont G, Bishop DS, Fortinsky RH (2011). A telephone-delivered psychosocial intervention improves dementia caregiver adjustment following nursing home placement. Int J Geriatr Psychiatry.

[12] Bank AL, Argüelles S, Rubert M, Eisdorfer C, Czaja SJ (2006). The value of telephone support groups among ethnically diverse caregivers of persons with dementia. Gerontologist.

[13] Mahoney DF (2010). An evidence-based adoption of technology model for remote monitoring of elders' daily activities. Ageing Int.

[14] Finkel S, Czaja SJ, Schulz R, Martinovich Z, Harris C, Pezzuto D (2007). E-care: A telecommunications technology intervention for family caregivers of dementia patients. Am J Geriatr Psychiatry.

[15] Moulin F, De Rotrou J, Batrancourt B, Cristancho-Lacroix V, Wrobel J, Legouverneur G, El Haj M, Rigaud AS, Broca Hospital Team (2011). Comprendre et agir ensemble pour retrouver mon équilibre.

[16] De Rotrou J, Thévenet S, Richard A, Cantegreil I, Wenisch E, Chausson C, Moulin F, Batouche F, Rigaud AS (2006). Impact of a psychoeducational program on stress of caregivers of Alzheimer disease patients. Encephale.

[17] De Rotrou J, Cantegreil I, Faucounau V, Wenisch E, Chausson C, Jegou D, Grabar S, Rigaud AS (2011). Do patients diagnosed with Alzheimer's disease benefit from a psycho-educational programme for family caregivers? A randomised controlled study. Int J Geriatr Psychiatry.

[18] American Psychiatric Association (2000). DSM-IV-TR. Diagnostic and statistical manual of mental disorders.

[19] McKhann G, Drachman D, Folstein M, Katzman R, Price D, Stadlan EM (1984). Clinical diagnosis of Alzheimer's disease: Report of the NINCDS-ADRDA Work Group under the auspices of Department of Health and Human Services Task Force on Alzheimer's Disease. Neurology.

[20] Cohen S, Kamarck T, Mermelstein R (1983). A global measure of perceived stress. J Health Soc Behav.

[21] Cristancho-Lacroix V, Kerherve H, Pino M, Legouverneur G, Rigaud A (2011). Usability assessment of a psycho-educational website for Alzheimer's disease caregivers. Alzheimer's & Dementia.

[22] Stetter F, Kupper S (2002). Autogenic training: A meta-analysis of clinical outcome studies. Appl Psychophysiol Biofeedback.

[23] Bernstein DA, Borkovec TD, Stevens HH (2000). A guidebook for helping professionals. New directions in progressive relaxation training.

[24] Bruchon-Schweitzer M (2002). Modèles, concepts et méthodes. Psychologie de la santé.

[25] Cole SR (1999). Assessment of differential item functioning in the Perceived Stress Scale-10. J Epidemiol Community Health.

[26] Steffen AM, McKibbin C, Zeiss AM, Gallagher-Thompson D, Bandura A (2002). The revised scale for caregiving self-efficacy: Reliability and validity studies. The Journals of Gerontology Series B: Psychological sciences and social sciences.

[27] Marziali E, Garcia LJ (2011). Dementia caregivers' responses to 2 Internet-based intervention programs. Am J Alzheimers Dis Other Demen.

[28] Teri L, Truax P, Logsdon R, Uomoto J, Zarit S, Vitaliano PP (1992). Assessment of behavioral problems in dementia: The revised memory and behavior problems checklist. Psychol Aging.

[29] Hébert R, Bravo G, Girouard D (2010). Fidélité de la traduction française de trois instruments d'évaluation des aidants naturels de malades déments. Can. J. Aging.

[30] Ankri J, Andrieu S, Beaufils B, Grand A, Henrard JC (2005). Beyond the global score of the Zarit Burden Interview: Useful dimensions for clinicians. Int J Geriatr Psychiatry.

[31] Beck AT, Steer RA, Brown GK (1996). Beck Depression Inventory–II.

[32] Bouvard M, Cottraux J (2010). Protocoles et échelles d'évaluation en psychiatrie et psychologie. Ed. Masson. Issy-les-Moulineaux; 5th edition.

[33] Bucquet D, Condon S, Ritchie K (1990). The French version of the Nottingham Health Profile. A comparison of items weights with those of the source version. Soc Sci Med.

[34] Folstein MF, Folstein SE, McHugh PR (1975). "Mini-mental state". A practical method for grading the cognitive state of patients for the clinician. J Psychiatr Res.

[35] Israël L, Guelfi J (1996). Evaluation de l’autonomie, des activités instrumentales de la vie quotidienne, IADL. L’évaluation clinique standardisée en psychiatrie.

[36] Telemedicine Technologies.

[37] ICHHT (2001). J Postgrad Med.

[38] Pedrelli P, Feldman GC, Vorono S, Fava M, Petersen T (2008). Dysfunctional attitudes and perceived stress predict depressive symptoms severity following antidepressant treatment in patients with chronic depression. Psychiatry Res.

[39] Braun V, Clarke V (2006). Using thematic analysis in psychology. Qualitative Research in Psychology.

[40] Kernisan LP, Sudore RL, Knight SJ (2010). Information-seeking at a caregiving website: A qualitative analysis. J Med Internet Res.

[41] Griffiths F, Lindenmeyer A, Powell J, Lowe P, Thorogood M (2006). Why are health care interventions delivered over the Internet? A systematic review of the published literature. J Med Internet Res.

[42] Gaugler JE, Mittelman MS, Hepburn K, Newcomer R (2009). Predictors of change in caregiver burden and depressive symptoms following nursing home admission. Psychol Aging.

[43] Siegert RJ, Jackson DM, Tennant A, Turner-Stokes L (2010). Factor analysis and Rasch analysis of the Zarit Burden Interview for acquired brain injury carer research. J Rehabil Med.

[44] Young C (2013). Community management that works: How to build and sustain a thriving online health community. J Med Internet Res.

[45] Dubreuil A, Hazif-Thomas C (2013). Les aidants et la santé sur Internet ou les «aidantnautes» s’entraident. NPG Neurologie - Psychiatrie - Gériatrie.

[46] Eysenbach G, CONSORT-EHEALTH Group (2011). CONSORT-EHEALTH: improving and standardizing evaluation reports of Web-based and mobile health interventions. J Med Internet Res.

