# Supplementary material for: Evaluating the Efficacy of a Web-Based Program (Diapason) for Informal Caregivers of Patients With Alzheimer’s Disease: Protocol for a Randomized Clinical Trial
Source: JMIR Res Protoc. 2013 Dec 6;2(2):e55. doi: 10.2196/resprot.2978 (PMC3869082; doi:10.2196/resprot.2978)
Supplement: Supplementary file 1 [file resprot_v2i2e55_app1.pdf]

CONSORT-EHEALTH Checklist V1.6.2 Report

(based on CONSORT-EHEALTH V1.6), available at [http://tinyurl.com/consort-ehealth-v1-6].

2978

Date completed

10/9/2013 16:32:07

by

Victoria Cristancho-Lacroix

Study protocol of a randomized clinical trial to evaluate the efficacy of a web-based program for informal caregivers of patients with Alzheimer's disease: Diapason project.

TITLE

1a-i) Identify the mode of delivery in the title

"Study protocol of a randomized clinical trial to evaluate the efficacy of a web-based program for informal caregivers of patients with Alzheimer's disease: Diapason project".

1a-ii) Non-web-based components or important co-interventions in title

1a-iii) Primary condition or target group in the title

Yes:
"Study protocol of a randomized clinical trial to evaluate the efficacy of a web-based program for informal caregivers of patients with Alzheimer's disease: Diapason project."

ABSTRACT

1b-i) Key features/functionalities/components of the intervention and comparator in the METHODS section of the ABSTRACT

"Methods and designs: In this protocol 80 informal caregivers of patients followed at Broca Hospital are recruited offline and randomized in the experimental condition (EC) or the control condition (CC). The volunteers in EC have to visit a closed online user group at least once a week and validate one new session of this full-automated web-program, during 12 weeks. Each week a new thematic is added to the website. The participants in the CC receive usual care, and have access to the Diapason program after their participation (6 months)."

1b-ii) Level of human involvement in the METHODS section of the ABSTRACT

In the abstract, methods and design: "...The volunteers in EC have to visit a closed online user group at least once a week and validate one new session of this fully automated web-program, during 12 weeks".

1b-iii) Open vs. closed, web-based (self-assessment) vs. face-to-face assessments in the METHODS section of the ABSTRACT

"In this study 80 informal caregivers of patients followed at Broca Hospital are recruited offline and randomized in the experimental condition (EC) ...".
In this study the assessments (visits) are conducted in face-to-face by an experienced psychologist in research protocols.
'Face-to-face evaluations for both groups are planned every 3 months (M0 – M3 and M6)."
"Assessing the efficacy of a French web-based psycho-educational program (called Diapason) with an unblinded randomized clinical trial was the aim of..."

1b-iv) RESULTS section in abstract must contain use data

It is an ongoing protocol. Results are not available yet.

1b-v) CONCLUSIONS/DISCUSSION in abstract for negative trials

INTRODUCTION

2a-i) Problem and the type of system/solution

"CGs are submitted to high levels of stress, and are at higher risk of weakening mental and physical health, lower life expectancy and lesser economic security than people who are not confronted to such stressful situations"

"There are many reasons for caregivers to use or to prefer a distance intervention instead of a face-to-face one.(...) Finally, some of them live in remote regions, and other CGs do not feel at ease with face-to-face interventions or prefer a flexible time/content intervention "

2a-ii) Scientific background, rationale: What is known about the (type of) system

"There are many reasons for caregivers to use or to prefer a distance intervention instead of a face-to-face one.(...) Finally, some of them live in remote regions, and other CGs do not feel at ease with face-to-face interventions or prefer a flexible time/content intervention [7]."

"Distance interventions, i.e. based on Information and Communications Technology (ICT) appeared in the earlier part of 21st century in order to propose an alternative intervention to caregivers unable to access health centers delivering face-to-face programs. Distant programs have shown a positive effect on self-perceived stress, burden, depression symptoms, and social support of caregivers.
In the case of caregivers of patients with dementia, several websites exist in France, but these programs have not been, to our knowledge, subjected to a randomized clinical trial."

METHODS

3a) CONSORT: Description of trial design (such as parallel, factorial) including allocation ratio

"The purpose of this article is to present the study protocol of a randomized clinical trial designed to evaluate the efficacy of Diapason, a web-based psycho-educational program for caregivers of patients with Alzheimer's disease (AD).
Our hypothesis is that the Diapason program reduces the caregiver's perceived stress and burden and enhances his/her self-efficacy and self-perceived health."

3b) CONSORT: Important changes to methods after trial commencement (such as eligibility criteria), with reasons

No, we did not change the method after trial commencement.

3b-i) Bug fixes, Downtimes, Content Changes

4a) CONSORT: Eligibility criteria for participants

"Eligible participants are informal French-speaking caregivers (family or not, providing care to the patient during 4 hours or more a week) of an AD patient diagnosed in the memory center of the Broca hospital, Paris, France, and who fulfills the criteria in the Diagnostic and Statistical Manual of Mental Disorders, 4th Edition (DSM-IV [18]) or National Institute of Neurological and Communicative Disorders and Stroke and the Alzheimer's disease and Related Disorders Association (NINCDS-ADRDA) criteria [19]. To be included in the trial, caregivers have to be aged 18 or over to be able to provide an informed consent, to score 12 or over on the Perceived Stress Scale of 14 items (PSS-14 [20]) during screening and to have a computer with an internet access at home with an email address regularly used. If participants (CGs) are on psychopharmacological treatment or therapy, they are required to keep the same treatment at least two months before inclusion in the protocol."

4a-i) Computer / Internet literacy

In elibility criteria: "...and to have a computer with an internet access at home
with an email address regularly used."

4a-ii) Open vs. closed, web-based vs. face-to-face assessments:

This is a closed face-to-face protocol:

"The participants are recruited either during the follow-up consultation of a patient a) the geriatrician/neurologist delivers the general information about the protocol and gives a contact form to fill in and drop off at the memory center's reception desk or b) the CGs fill in the contact form available in the waiting room and drop it off at the memory center's reception desk. One of the two research psychologists previously trained in the protocol

4a-iii) Information giving during recruitment

4b) CONSORT: Settings and locations where the data were collected

"Data are collected via an electronic case-report form (e-CRF) are centralized
and store on a secured server using "CleanWEB" system "

4b-i) Report if outcomes were (self-)assessed through online questionnaires

"When the participant delivers a positive answer, the first visit (M0) at the hospital is scheduled together with the psychologist. The duration of each visit (M0-M3 and M6) is estimated to 90 minutes."

"Satisfaction towards the program content (weekly paper-based survey filled at home)"

"6. The participants randomized in the EC receive the material (weekly paper – based survey, a journey book and a user's manual of the website)..."

4b-ii) Report how institutional affiliations are displayed

5) CONSORT: Describe the interventions for each group with sufficient details to allow replication, including how and when they were actually administered

5-i) Mention names, credential, affiliations of the developers, sponsors, and owners

5-ii) Describe the history/development process

5-iii) Revisions and updating

5-iv) Quality assurance methods

5-v) Ensure replicability by publishing the source code, and/or providing screenshots/screen-capture video, and/or providing flowcharts of the algorithms used

5-vi) Digital preservation
Yes it does:
"Broca Hospital Team, Moulin F, De Rotrou J, Batrancourt B, Cristancho-Lacroix V, Wrobel J, Legouverneur G, El Haj M, Rigaud AS. Website Diapason: Comprendre et agir ensemble pour retrouver mon équilibre 2011. http://www.etreaudiapason.com/. www.webcitation.org/6JtmmPcHq"
5-vii) Access
"Diapason is a free password-protected web-site. The program is run in twelve thematic weekly sessions organized in the following order:"

"The participants are recruited either during the follow-up consultation of a patient a) the geriatrician/neurologist delivers the general information about the protocol and gives a contact form to fill in and drop off at the memory center's reception desk or b) the CGs fill in the contact form available in the waiting room and drop it off at the memory center's reception desk.One of the two research psychologists previously trained in the protocol contacts the caregiver, checks his/her eligibility criteria and explains the benefits, constraints, and schedule of the protocol. The psychologist gives an information notice to the caregiver and proposes to contact him/her a few days later. If the caregiver agrees with the protocol and meets the criteria for inclusion, the screening session (M0) is scheduled with the caregiver."
5-viii) Mode of delivery, features/functionalities/components of the intervention and comparator, and the theoretical framework
"Diapason is a web-based version of a psycho-educational program, inspired by the group intervention sessions from the geriatric service of Broca Hospital called AIDMA program. AIDMA was assessed in a previous study including 167 dyads "patient-caregiver" and showed a significant improvement in disease understanding and in ability to cope with care-recipients' disease."

"The Diapason program is an adapted computerized version of a psycho-educational program -AIDMA- created by the Geriatric Service of Broca Hospital.

Diapason is a free password-protected web-site. The program is run in twelve thematic weekly sessions organized in the following order"
5-ix) Describe use parameters

5-x) Clarify the level of human involvement

5-xi) Report any prompts/reminders used
The website offer an online feedback (at the website). It indicates to each participant the number of session(s) that should be validated.

We use external reminders only when the participant is clearly late to validate the sessions (3 weeks or more). In these cases the participant is called by a psychologist.

Finally, the psychologist calls each participant to remind him/her the next appointment some days before the visit.
5-xii) Describe any co-interventions (incl. training/support)
Both groups, experimental and control "receive usual care. It consists in a geriatric semi-annual follow-up appointment during which the caregiver obtains illness information from the geriatrician. (...)Every participant (A/N. in both groups) is advised to look for more specific help (i.e. that of a psychologist or a physician) when he/she feels it necessary and then to report it to the main investigator."
6a) CONSORT: Completely defined pre-specified primary and secondary outcome measures, including how and when they were assessed

"The duration of each visit (M0-M3 and M6) is estimated to 90 minutes. The baseline visit is usually conducted as follows:
1.The research psychologist answers the questions on information notice and the participant signs the informed consent if he/she agrees.
2.Evaluation with PSS-14 (primary outcome).
3.Randomization if PPS-14 total score is 12 or over.
4.Demographical interview and control questions of caregiver’s and patient’s variables.
5.Assessment of secondary variables (researcher-administered, and then self-administered surveys).
6.The participants randomized in the EC receive the material (weekly paper –based survey, a journey book and a user’s manual of the website) and a personal access-code to the website. Then, they are trained on how to use the web-based program.
7.The CC participants are notified that they will receive a website access at the end of their participation to this protocol (six months after M0).
8.Planning follow-up visits (M3 – M6).
For the CC and EC groups, the assessments at M3 and M6 visits are similar, and go as follows: first, evaluation of caregiver variables (time spent on caregiving, use of respite resources, stressful events, etc.) and patient status (hospitalization or other unexpected event occurred in the last three months). Then, measurements with self-administered scales or administered by an interviewer. The measures used in this RCT are summarized in Table 1. "

6a-i) Online questionnaires: describe if they were validated for online use and apply CHERRIES items to describe how the questionnaires were designed/deployed

6a-ii) Describe whether and how “use” (including intensity of use/dosage) was defined/measured/monitored

Yes, we will obtain the website statistics of use by participant and by period (M0-M3/ M3-M6).

6a-iii) Describe whether, how, and when qualitative feedback from participants was obtained

6b) CONSORT: Any changes to trial outcomes after the trial commenced, with reasons

Not, we did not.

7a) CONSORT: How sample size was determined

7a-i) Describe whether and how expected attrition was taken into account when calculating the sample size

7b) CONSORT: When applicable, explanation of any interim analyses and stopping guidelines

N/A

8a) CONSORT: Method used to generate the random allocation sequence

"Randomization:
A computer-generated randomization list is used to assign the participants in the experimental condition (EC) group or in the control condition (CC) group after assessment with PSS-14 and all the inclusion and non-inclusion criteria checked."

8b) CONSORT: Type of randomisation; details of any restriction (such as blocking and block size)

"Blocking and stratification by gender and relationship (spouses vs. non spouses) were used to generate the randomization list."

9) CONSORT: Mechanism used to implement the random allocation sequence (such as sequentially numbered containers), describing any steps taken to conceal the sequence until interventions were assigned

"A computer-generated randomization list is used to assign the participants in the experimental condition (EC) group or in the control condition (CC) group after assessment with PSS-14 and all the inclusion and non-inclusion criteria checked."

10) CONSORT: Who generated the random allocation sequence, who enrolled participants, and who assigned participants to interventions

The random allocation list is computer-generated.
The participants are enrolled by the physicians.
The participants are assigned to the interventions by a trained research psychologist who also conducts the evaluations:
"One of the two research psychologists previously trained in the protocol contacts the caregiver, checks his/her eligibility criteria and explains the benefits, constraints, and schedule of the protocol. The psychologist gives an information notice to the caregiver and proposes to contact him/her a few days later. If the caregiver agrees with the protocol and meets the criteria for inclusion, the screening session (M0) is scheduled with the caregiver."

11a) CONSORT: Blinding - If done, who was blinded after assignment to interventions (for example, participants, care providers, those assessing outcomes) and how

11a-i) Specify who was blinded, and who wasn't

In this protocol it was not possible to conduct a blind evaluation.
Indeed, at the end of the first visit M0, the assessor give to the participants randomized in the experimental group a training about how to use the website, then shows how to fulfill a paper-based questionnaire about their satisfaction on the program. In addition, the assessors have to control the assiduity of experimental group on the website.

11a-ii) Discuss e.g., whether participants knew which intervention was the "intervention of interest" and which one was the "comparator"

11b) CONSORT: If relevant, description of the similarity of interventions

Not relevant for this study.

12a) CONSORT: Statistical methods used to compare groups for primary and secondary outcomes

"A description of the characteristics of the two groups will be performed using percentages for categorical variables and means with standard deviation for quantitative variables. For primary and secondary outcomes, student t-tests or Wilcoxon test if required, as well as covariance analysis to take the regression to the mean into account, will be used to compare means between experimental and control groups. Percentages will be compared using Chi-Square test, or Fisher's exact test if required. Calculations will be performed using SAS software. "

12a-i) Imputation techniques to deal with attrition / missing values

"All the analyses will be conducted according to the intention to treat principle and to handle with missing data, multiple imputation will be used if the missing at random or missing completely at random hypothesis holds."

12b) CONSORT: Methods for additional analyses, such as subgroup analyses and adjusted analyses

"Percentages will be compared using Chi-Square test, or Fisher's exact test if required."

RESULTS

13a) CONSORT: For each group, the numbers of participants who were randomly assigned, received intended treatment, and were analysed for the primary outcome

It is an ongoing protocol. Results are not available yet.

13b) CONSORT: For each group, losses and exclusions after randomisation, together with reasons

It is an ongoing protocol. Results are not available yet.

13b-i) Attrition diagram

14a) CONSORT: Dates defining the periods of recruitment and follow-up

"The enrollment began in October 2011, and participants currently recruited will finish their evaluations in January 2014. The results are expected for June 2014."

14a-i) Indicate if critical "secular events" fell into the study period

14b) CONSORT: Why the trial ended or was stopped (early)

N/A. It is an ongoing protocol.

15) CONSORT: A table showing baseline demographic and clinical characteristics for each group

It is an ongoing protocol. The results are expected for June 2014.

15-i) Report demographics associated with digital divide issues

It is an ongoing protocol. This item is not available yet.

16a) CONSORT: For each group, number of participants (denominator) included in each analysis and whether the analysis was by original assigned groups

16-i) Report multiple "denominators" and provide definitions

It is an ongoing protocol. This item is not available yet.

16-ii) Primary analysis should be intent-to-treat

17a) CONSORT: For each primary and secondary outcome, results for each group, and the estimated effect size and its precision (such as 95% confidence interval)

It is an ongoing protocol. This item is not available yet.

17a-i) Presentation of process outcomes such as metrics of use and intensity of use

17b) CONSORT: For binary outcomes, presentation of both absolute and relative effect sizes is recommended

It is an ongoing protocol. This item is not available yet.

18) CONSORT: Results of any other analyses performed, including subgroup analyses and adjusted analyses, distinguishing pre-specified from exploratory

It is an ongoing protocol. This item is not available yet.

18-i) Subgroup analysis of comparing only users

19) CONSORT: All important harms or unintended effects in each group

It is an ongoing protocol. This item is not available yet.

19-i) Include privacy breaches, technical problems

19-ii) Include qualitative feedback from participants or observations from staff/researchers

DISCUSSION

20) CONSORT: Trial limitations, addressing sources of potential bias, imprecision, multiplicity of analyses

20-i) Typical limitations in ehealth trials

The limitations of this trial:
- It is not blinded.
- The assessments for the baseline and the follow-ups at the hospital reduce the pertinence of this web-based program for some of volunteers who cannot come to the visits.
- Probably an individual support via the website added to this program would improve its impact.

21) CONSORT: Generalisability (external validity, applicability) of the trial findings

21-i) Generalizability to other populations

It is an ongoing protocol. This item is not available yet.

21-ii) Discuss if there were elements in the RCT that would be different in a routine application setting

22) CONSORT: Interpretation consistent with results, balancing benefits and harms, and considering other relevant evidence

22-i) Restate study questions and summarize the answers suggested by the data, starting with primary outcomes and process outcomes (use)

It is an ongoing protocol. This item is not available yet.

22-ii) Highlight unanswered new questions, suggest future research

Other information

23) CONSORT: Registration number and name of trial registry

Trial registration: NCT01430286 - ID: P081002 -
It is available at the next link: http://clinicaltrials.gov/ct2/show/NCT01430286?term=diapason&rank=1

24) CONSORT: Where the full trial protocol can be accessed, if available

At the page of clinical-trials.gov: http://clinicaltrials.gov/ct2/show/NCT01430286?term=diapason&rank=1
or by contact with the first author: victoria.cristancho@gmail.com

25) CONSORT: Sources of funding and other support (such as supply of drugs), role of funders

"This study was partially funded by French Health Minister (Projet de Recherche en Qualité Hospitalière 2009 – PREQHOS 2009) and by “Fondation Méderic Alzheimer” project grant 2012-2014. It is monitored by the Clinical Research Unit of Cochin Hospital (URC/CIC Necker Cochin - Under the direction of Jean-Marc Tréluyer)."

X26-i) Comment on ethics committee approval

"This study protocol was submitted to the French ethical Committee for Persons’ Protection (CPP) and received approval on July 2011. Before they enter the study, all participants receive an information sheet and sign a written consent form.
The study provides equal opportunity to access the program. Caregivers who do not meet the inclusion criteria can access the website and program as external participants. Also, every participant is asked to search another form of help (i.e. that of a psychologist or a physician) if he/she feels the need to, and to report it to the main investigator."

x26-ii) Outline informed consent procedures

X26-iii) Safety and security procedures

X27-i) State the relation of the study team towards the system being evaluated

"The authors declare that they have no competing interests."
